# Bioreactor-based bioremediation of hydrocarbon-polluted Niger Delta marine sediment, Nigeria

**DOI:** 10.1007/s13205-011-0030-8

**Published:** 2011-10-21

**Authors:** Chioma Blaise Chikere, Blaise Ositadinma Chikere, Gideon Chijioke Okpokwasili

**Affiliations:** 1 Department of Microbiology, University of Port-Harcourt, P.M.B. 5323, East-West Road, Choba, Port Harcourt, Rivers State Nigeria; 2 Health, Safety and Environment (HSE), Shell Petroleum Development Company, P.O. Box 263, Port Harcourt, Rivers State Nigeria

**Keywords:** Niger Delta, Marine sediment, Bioreactor, Crude oil, Bonny loading jetty

## Abstract

Crude oil-polluted marine sediment from Bonny River loading jetty Port Harcourt, Nigeria was treated in seven 2.5 l stirred-tank bioreactors designated BNPK, BNK5, BPD, BNO_3_, BUNa, BAUT, and BUK over a 56-day period. Five bioreactors were biostimulated with either K_2_HPO_4_, NH_4_NO_3_, (NH_4_)_2_SO_4_, NPK, urea or poultry droppings while unamended (BUNa) and heat-killed (BAUT) treatments were controls. For each bioreactor, 1 kg (wet weight) sediment amended with 1 l seawater were spiked with 20 ml and 20 mg of crude oil and anthracene which gave a total petroleum hydrocarbons (TPH) range of 106.4–116 ppm on day 0. Polycyclic aromatic hydrocarbons (PAH) in all spiked sediment slurry ranged from 96.6 to 104.4 ppm. TPH in each treatment was ≤14.9 ppm while PAH was ≤6.8 ppm by day 56. Treatment BNO_3_ recorded highest heterotrophic bacterial count (9.8 × 10^8^ cfu/g) and hydrocarbon utilizers (1.15 × 10^8^ cfu/g). By day 56, the percentages of biodegradation of PAHs, as measured with GC–FID were BNK5 (97.93%), BNPK (98.38%), BUK (98.82%), BUNa (98.13%), BAUT (93.08%), BPD (98.92%), and BNO_3_ (98.02%). BPD gave the highest degradation rate for PAH. TPH degradation rates were as follows: BNK5 (94.50%), BNPK (94.77%), BUK (94.10%), BUNa (94.77%), BAUT (75.04%), BPD (95.35%), BNO_3_ (95.54%). Fifty-six hydrocarbon utilizing bacterial isolates obtained were *Micrococcus* spp. 5 (9.62%), *Staphylococcus* spp. 3 (5.78%), *Pseudomonas* spp. 7 (13.46%), *Citrobacter* sp. 1 (1.92%), *Klebsiella* sp. 1 (1.92%), *Corynebacterium* spp. 5 (9.62%), *Bacillus* spp. 5 (9.62%), *Rhodococcus* spp. 7 (13.46%), *Alcanivorax* spp. 7 (13.46%), *Alcaligenes* sp. 1 (1.92%), *Serratia* spp. 2 (3.85%), *Arthrobacter* spp. 7 (13.46%), *Nocardia* spp. 2 (3.85%), *Flavobacterium* sp. 1 (1.92%), *Escherichia* sp. 1 (1.92%), *Acinetobacter* sp. 1 (1.92%), *Proteus* sp. 1 (1.92%) and unidentified bacteria 10 (17%). These results indicate that the marine sediment investigated is amenable to bioreactor-based bioremediation and that abiotic factors also could contribute to hydrocarbon attenuation as recorded in the heat-killed (BAUT) control.

## Introduction

Aquatic ecosystems are permanently challenged with hydrocarbons of different composition and origin. During exploration, production, refining, transport and storage of petroleum and petroleum products, some accidental spills could occur (Mnif et al. 2009). The threat of petroleum pollution not only from natural sources such as seeps but also by anthropogenic activities as spillages during transportation, direct discharge from effluent treatment plants and other emissions, endangers the marine biodiversity (Gertler et al. 2009a; Nogales et al. 2011). For instance, in Nigeria, the Niger Delta region produces more than 80% of the country’s crude oil. There is presently an unprecedented increase in the upstream and downstream activities of the oil and allied industries in this oil-rich area (Abu and Chikere 2006; Chikere et al. 2009a, b). Over the years, these oil companies have generated myriad of pollutants in the form of gaseous emissions, oil spills, effluents and solid waste (Odeyemi and Ogunseitan 1985; Nweke and Okpokwasili 2004) that have polluted the marine environment beyond sustainability. Heightened navigational activities in inland and coastal waters of the Niger Delta region is another anthropogenic source of refined petroleum pollution of the aquatic environment. An investigation of the polycyclic aromatic hydrocarbons (PAHs) concentrations in some Niger Delta sediments carried out by Ezemonye and Ezemonye (2005) revealed elevated values of these priority pollutants in the sediments studied.

Given the high energy content of highly reduced compounds like petroleum hydrocarbons, it is hardly surprising that many microbes have evolved or acquired the ability to utilize hydrocarbons as sources of carbon and energy (Yakimov et al. 2007; Gertler et al. 2009b). The biodegradation of hydrocarbons is a process well established in nature and known to man for a long time. Mostly limited due to the low mineral nutrient levels in seawater and sediments, biodegradation of hydrocarbons is mediated by numerous genera of marine bacteria (Head and Swannell 1999; Kasai et al. 2002; Head et al. 2006; Paisse et al. 2008). Knowledge of indigenous oil-degrading bacteria and their nutritional requirements have helped scientists to look for ways of employing self-purification/cleaning function of the aquatic ecosystem in order to mitigate marine oil pollution by bioremediation. Bioremediation is the biotechnology which makes use of the catabolic activities of indigenous hydrocarbon utilizing bacteria to decontaminate oil-polluted environments (Mahmoud et al. 2009). Bioremediation can be applied as green technologies as it offers an environmentally friendly and cost effective response to marine oil pollution. Three principal approaches of this technique: natural attenuation (reliance on natural biodegradation activities and rates), which is sometimes called intrinsic bioremediation; biostimulation (stimulation of natural activities by environmental modification such as fertilizer addition to increase rates of biodegradation); and bioaugmentation (addition of exogenous microorganisms to supplant the natural degradative capacity of the hydrocarbon-impacted ecosystem) for in situ biodegradation have been applied several times at pilot and field scale levels with varying degrees of success (Kaplan and Kitts 2004; Prince and Atlas 2005; Chikere et al. 2009a, b; Gertler et al. 2009a).

Based on the different bioremediation approaches mentioned above, several biological methods are employed in the treatment of petroleum impacted environmental media which include bioreactor-based treatment, landfarming, biopiling, composting, bioventing, biosparging, biofiltration and phytoremediation (rhizoremediation) (Young and Cerniglia 1995; Siciliano et al. 2003; Montiel et al. 2009). Of all these, bioreactor-based treatment has an edge over other methods because it provides an optimal controlled environment for the biodegradation of hydrocarbon-polluted media and eliminates most of the rate-limiting/variable factors such as oxygen supply, optimal pH, temperature and specific nutrient formulations associated with the other methods (Van Hamme et al. 2003). Bioreactors, which can be applied in bioremediation strategies, are basically tanks in which living organisms carry out biological reactions. Their efficiency is based on the ability of bacteria to attach to inert packing, such as granular activated carbon, at interfaces to generate high biomass (Bouwer and McCarty 1982; Teitzel and Parsek 2003). The reactor should also be easy to maintain and operate (Evangelho et al. 2001), and should be able to function under aerobic and anaerobic conditions. Bioreactors can accommodate solids concentrations of 5–50% wt/vol. Through break up of solid aggregates and dispersion of insoluble substrates, hydrocarbon desorption and contact with the aqueous phase is promoted, resulting in increased biodegradation. Bioreactor-based petroleum sludge/slurry treatment also allows management of volatile organic compounds (VOCs) by creating reactor conditions which accelerate the process of bioremediation of these VOCs rather than their attenuation via volatilization as obtained in other open treatment methods (Young and Cerniglia 1995).

Various types of bioreactors are widely used in a large variety of aerobic bioprocesses such as aerobic fermentation, biological waste water and hydrocarbon impacted soil/sediments treatments among others (Van Hamme et al. 2003). Stirred tank bioreactors are mechanically agitated where the stirrers are the main gas-dispersing tools and provide high values of mass transfer rates coupled with excellent mixing. Pneumatically agitated bioreactors have two configurations namely bubble columns and airlift bioreactors. In these bioreactors, the low shear environment compared to the stirred tanks is beneficial for successful cultivation of shear sensitive and filamentous cells (Garcia-Ochoa and Gomez 2009).

In the present research, 7 stirred tank bioreactors were used for the bioremediation of marine sediments impacted with petroleum hydrocarbons (crude oil and anthracene). Different nutrient regimens were formulated using organic and inorganic nutrient sources namely NPK fertilizer, urea fertilizer and poultry litter to enhance the biodegradation of the pollutants by the extant autochthonous marine hydrocarbon degrading bacteria. The objectives of the research were to use laboratory bioreactors to investigate the catabolic potential of natural marine microbial communities to biodegrade target hydrocarbon pollutants and also to evaluate the efficacy of biostimulation during hydrocarbon degradation by natural microbial communities augmented with nitrogen and phosphorus additions.

## Materials and methods

### Sampling site/sample collection

**Fig. 1:**
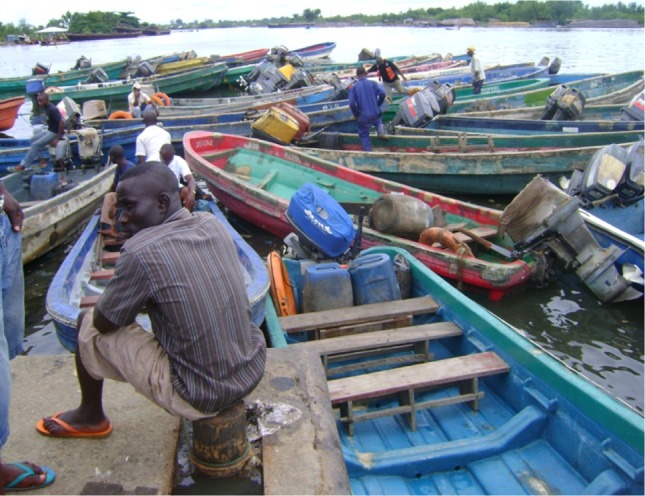
Speed boats and operators at Bonny Island loading jetty. These speed boats are driven with either premium motor spirit (PMS) or automotive gas oil (AGO), all refined petroleum

The sediments were collected from Bonny River loading jetty, in Bonny Island, Rivers State, Nigeria. Bonny Island is located in the South region of Nigeria and forms heart of the oil-rich Niger Delta. It houses Nigeria’s major crude oil export terminal and most of the country’s oil installations. This area also experiences heightened navigational activities and as such spills of petroleum hydrocarbons from both crude oil and refined products occur regularly. A section of speed boats and operators seen as at the time the sediments were collected are shown in Fig. 1. Films of petroleum products were seen on the surface of the seawater indicating that this marine ecosystem is constantly exposed to petroleum hydrocarbons. Sediment samples were collected from a depth of 30 m with Eckman grab (Wild Life Supply Co., NY) with a sterile Thermocool warmer. Seawater was collected with sterile 20 l container. All samples were transported to the laboratory within 6 h for analyses.

### Bioslurry bioreactors

**Fig. 2:**
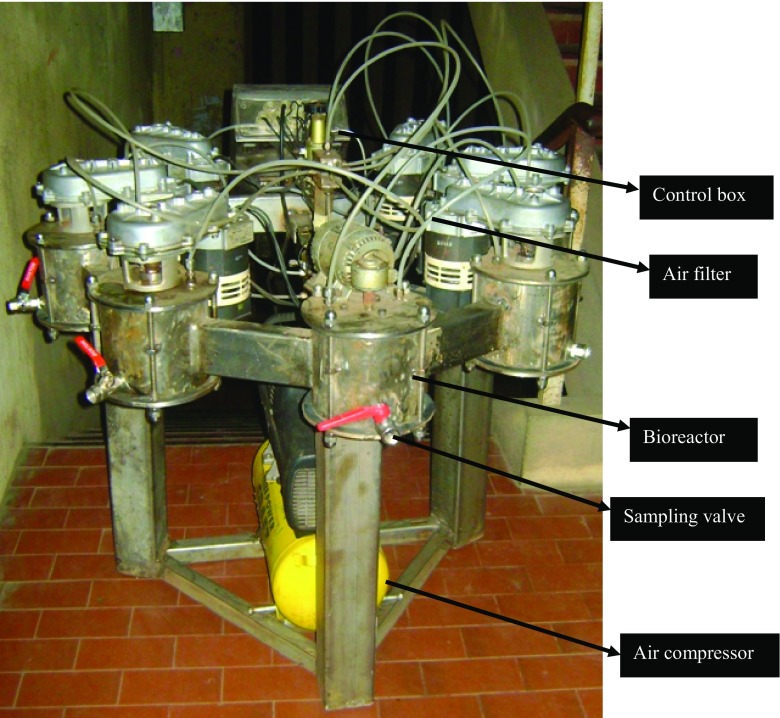
The 7 (2.5 l) bioreactors used in the bioremediation experiment

Bioremediation of hydrocarbon-impacted marine sediments from Bonny Island loading jetty was conducted with (7) 2.5 l bioslurry bioreactors (Fig. 2) operated over a 56-day period. Two reactors served as controls (unamended [designated BUNa] and heat-killed [designated BAUT]), while the remaining 5 served as nutrient-amended bioreactors. Each of the 7 bioreactors received 1 kg (wet weight) of sediments, 1 l of seawater, 20 ml of crude oil and 20 mg of anthracene (Table 1). For the controls, the unamended treatment was only spiked with the hydrocarbons without nutrient addition to determine whether the indigenous bacteria in the sediments have the natural propensity to degrade petroleum hydrocarbons where as the heat-killed treatment (killed by autoclaving sediments and seawater at 121 °C for 15 min at 15 psi on 2 consecutive days) was set up to measure the role of abiotic factors in the loss of petroleum hydrocarbons.

**Table 1:** Experimental design

Bioreactor code	Test experiment (amended)	Bioreactor code	Control experiment (unamended)
BNK5	1 kg of sediment + 1 l of seawater + 0.2 g PAH (anthracene) + 10 g (NH)_2_SO_4_ + 2 g K_2_HPO_4_ + 20 ml of crude oil	BUNa	1 kg of sediment + 1 l of seawater + 20 ml of crude oil + 0.2 g PAH (anthracene)
BPD	1 kg of sediment + 1 l of seawater + 0.2 g PAH (anthracene) + 20 ml of crude oil + 20 g of poultry droppings + 2 g K_2_HPO_4_	BAUT	1 kg of heat killed sediment + 1 l of heat killed seawater + 20 ml of crude oil + 0.2 g PAH (anthracene)
BUK	1 kg of sediment + 1 l of seawater + 20 ml of crude oil + 0.2 g PAH (anthracene) + 1 g K_2_HPO_4_ + 10 g of urea		
BNO_3_	1 kg of sediment + 1 l of seawater + 0.2 g PAH (anthracene) + 20 ml of crude oil + 10 g of NH_4_NO_3_ + 2 g of K_2_HPO_4_		
BNPK	1 kg of sediment + 1 l of seawater + 0.2 g PAH (anthracene) + 20 ml of crude oil + 20 g of NPK 20:10:10		

The bioreactors were loaded with sediment and hydrocarbons (crude oil and anthracene) and five were amended with nutrients while two served as controls as shown in Table 1.

**Fig. 3:**
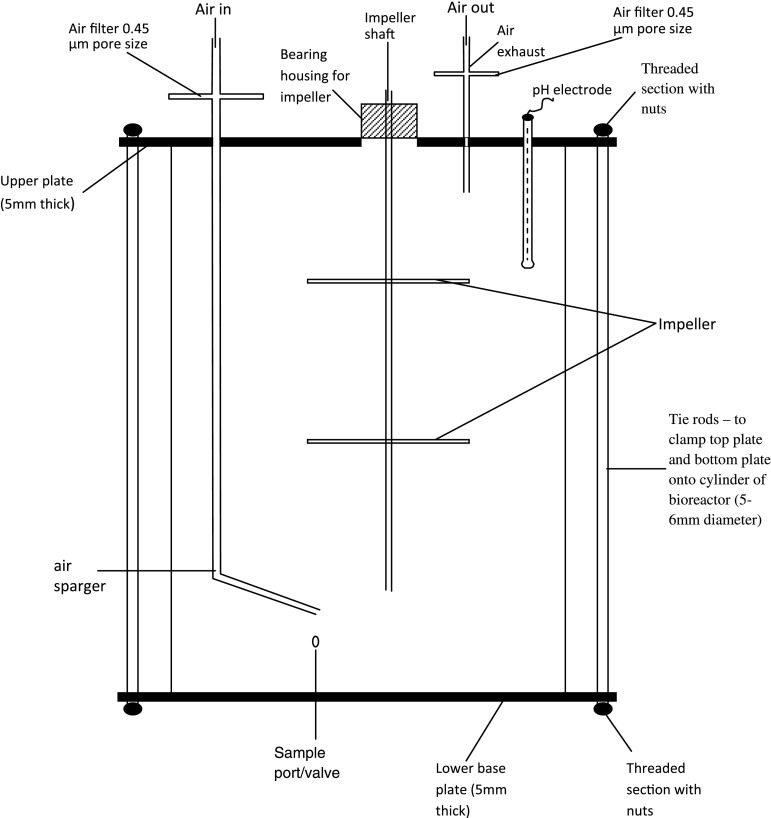
Design of the interior of the 2.5 l bioreactors used for the bioremediation experiment

The bioreactors were continuously stirred (by 2 impellers) at 150 rpm throughout the 56-day experimental period. The interior of the bioreactors with the accessories are shown in Fig. 3. Filtered air was supplied to the bioreactors from the air compressor through hoses running in and out of them. They were sealed with Teflon to prevent the ingress of atmospheric air and egress of the slurry and were operated at room temperature (28 °C) through out the experimental period. pH in the 7 bioreactors at day zero ranged from 7.3 to 7.9 after adjustment.

### Statistical analysis of data

Statistical analysis was performed on the data generated from the bacterial counts and hydrocarbon concentrations for the different treatments using one way ANOVA and Tukey’s Multiple Comparison Test. The software GraphPad Prism (GraphPad Software, CA, USA) for Windows version 5.01 was used to do the analysis.

### Enumeration/identification of total heterotrophic bacteria (THB) and hydrocarbon utilizing bacteria (HUB)

Bacterial counts for THB and HUB were done on days 0, 7, 14, 28 and 56, respectively. From each bioreactor, 1 g (wet weight) of sediment was homogenized in 0.85% of normal saline. Decimal dilutions (tenfold) of the suspensions were plated out in duplicate on Plate Count Agar (Merck, Germany) modified with 10% NaCl and incubated at 30 °C for 24 h for the THB counts. For HUB counts, appropriate dilutions of sediment suspensions from each bioreactor (1 g wet weight of sediment homogenized in 0.85% of normal saline) were plated out in duplicate on Bushnell-Haas agar (Sigma-Aldrich, USA) modified with 10% NaCl. Hydrocarbons were supplied through the vapour phase to putative hydrocarbon utilizers by placing sterile Whatmann No. 1 filter papers impregnated with 5 ml Okono medium crude oil in the lids of the inverted Petri plates. Plates were incubated at 30 °C for 7 days. Individual colonies of “putative” hydrocarbon utilizers were be picked off the Bushnell-Haas agar plates and subcultured in order to check their ability to utilize hydrocarbons by plating out again on Bushnell-Haas agar (Sigma-Aldrich, USA). Hydrocarbons were supplied to the colonies by the vapour phase transfer using crude oil. The following biochemical tests: oxidase, citrate utilization, catalase, indole production, triple sugar iron utilization, methyl red–Voges Proskauer, glucose fermentation, gelatin liquefaction, urease production were used to identify and characterize the hydrocarbon utilizing bacteria. Other phenotypic tests carried out were Gram stain and motility test. Antibiogram of all the Gram-negative bacilli was determined using the disc diffusion method (Chikere et al. 2008) with the following antibiotics: ampiclox, cotrimoxazole, gentamycin, nalidixic acid, chloramphenicol, nitrofurantoin, streptomycin, tetracycline and erythromycin to aid in the identification of *Alcanivorax* spp. as adapted from Wu et al. (2009). The disappearance of TPHs and PAHs was analyzed on each sampling day with GC–FID. The hydrocarbons in the sediment samples for each treatment in the bioreactors were quantified using an Agilent 6890N Network gas chromatograph equipped with flame ionization detector. The carrier gas was helium and the column with catalogue number HP-5(19091J-413) had the following dimensions: 30 m × 0.32 mm × 0.25 μm. Detector temperature was 350 °C, hydrogen gas flow rate was 35 ml/min, air flow rate was 350 ml/min, while helium gas flow rate was 20 ml/min. The inlet which was of electronic pneumatic capture splitless make was operated thus: temperature (275 °C); pressure (psi) 14.8; split flow rate (6.8 ml/min); total flow rate (25.8 ml/min). The initial and final temperatures of the oven were 65 and 325 °C, respectively. The run time was approximately 53.5 min, pressure was 14.8 psi while flow rate was 3.3 ml/min. All analyses were conducted in triplicates.

## Results

### Baseline characteristics of sediment sample

The values of the baseline bacterial counts (total heterotrophic and hydrocarbon utilizing bacteria), physicochemical parameters (pH, nitrate, phosphate, potassium, conductivity and total organic carbon contents) and gas chromatographic analysis of total petroleum hydrocarbons (TPH) and polycyclic aromatic hydrocarbons (PAHs) in the sediment sample are presented in Table 2. The bacterial counts (for both total heterotrophic and hydrocarbon utilizing bacteria) were within the same range of 10^5^ cfu/g which was indicative of the fact that the bacterial community making up the total heterotrophic bacteria were all capable of utilizing petroleum hydrocarbons. This phenomenon occurs when an environment is chronically exposed to hydrocarbons from anthropogenic sources (Rosenberg and Ron 1996; Yakimov et al. 2007; Gertler et al. 2009a, b). The concentrations of the TPH and PAHs in the sediment also showed that there is a metabolically active bacterial community in the sediments that probably uses the hydrocarbons as source of carbon and energy owing to their low concentration in this sediment that is always inundated with petroleum hydrocarbons. The baseline hydrocarbon contents in the sediment prior to bioremediation were 3.34 ppm and <0.1 ppm TPH and PAHs, respectively. The Okono medium crude oil sample used in the spiking of the sediment contained 606,863 and 8,748 ppm for both TPH and PAHs, respectively. All other parameters measured showed that there is active microbial activity in the sediment since their concentrations were low.

**Table 2:** Baseline characteristics of sediment sample

Parameter	Concentration
Total heterotrophic bacterial count (THB)	6.5 × 10^5^ cfu/g
Hydrocarbon utilizing bacterial count (HUB)	7.8 × 10^5^ cfu/g
pH	9.84
Conductivity	1,082 μS/cm
Potassium	18.7 mg/kg
Phosphate	1.65 mg/kg
Total organic carbon (TOC)	0.2%
Nitrate	2.65 mg/kg
Total petroleum hydrocarbons (TPH)	3.34 ppm
Polycyclic aromatic hydrocarbons (PAHs)	<0.1 ppm

### Bacterial counts and hydrocarbon degradation during bioremediation

During the 56-day bioremediation project, different trends were observed in all the biological and physicochemical parameters analyzed in the different amended and control sediment samples in the bioreactors.

**Fig. 4:**
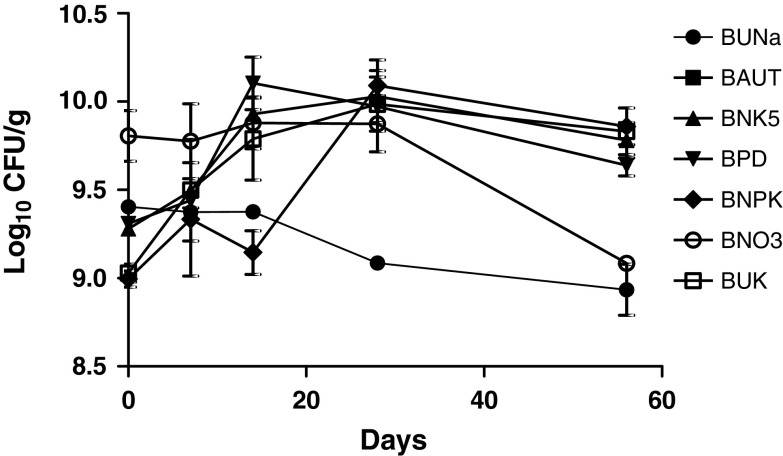
Total heterotrophic bacterial (THB) counts during the 56-day bioremediation

**Fig. 5:**
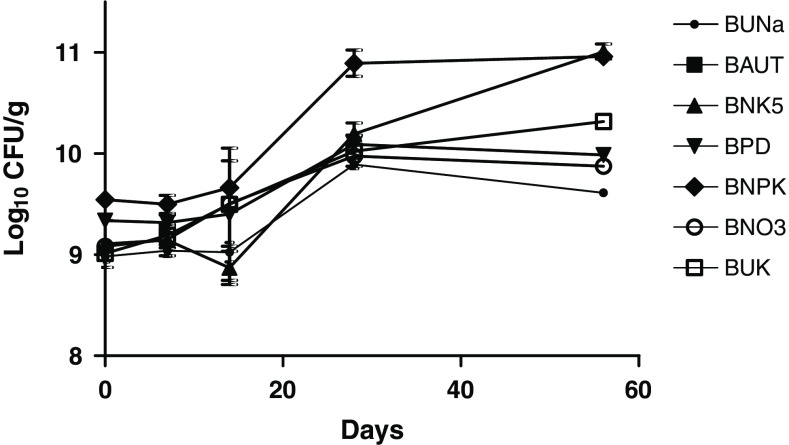
Hydrocarbon utilizing bacterial (HUB) counts during the 56-day bioremediation

Figure 4 shows the total heterotrophic bacterial counts (THB). There was a general increase for all treatments but BNO_3_ had the highest count of 7.9 × 10^9^ cfu/g. All other treatments which were BUNa, BNK5 BPD, BNPK and BUK increased from 10^8^ cfu/g by day 0 to 10^9^ cfu/g by day 56 when the experiment ended. BAUT recorded no bacterial growth throughout the experimental period. THB counts were statistically significant at *P* < 0.05 using one way ANOVA and Tukey’s multiple comparison test. Figure 5 represents the hydrocarbon utilizing bacterial (HUB) counts across all treatments including controls during the 56-day bioremediation. HUB counts in all treatments BNO_3_, BUNa, BNK5 BPD, BNPK and BUK increased from 10^8^ cfu/g by day 0 to 10^10^ cfu/g by day 56. BNPK recorded the highest HUB counts throughout the experimental period with a peak of 8.2 × 10^10^ cfu/g by day 35. The heat-killed control BAUT showed no growth for THB and HUB throughout the study period. HUB counts were statistically significant at *P* < 0.05 using one way ANOVA and Tukey’s multiple comparison test.

**Fig. 6:**
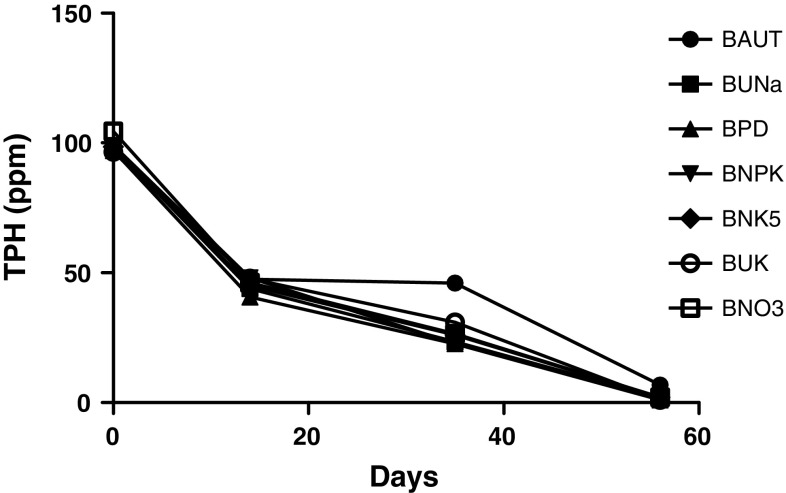
TPH content in different treatments during the 56-day bioremediation

**Fig. 7:**
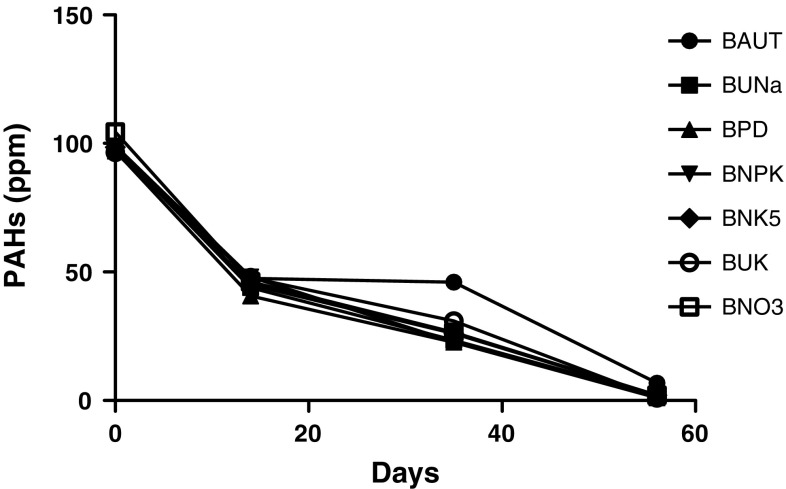
PAHs content in different treatments during the 56-day bioremediation

The degradation of the hydrocarbons (TPHs and PAHs) present in the sediment samples amended with different nutrient sources and the biotic and abiotic controls (BUNa and BAUT) are shown in Figs. 6 and 7. By day 56, the percentages of biodegradation of PAHs, as measured with GC–FID were BNK5 (97.93%), BNPK (98.38%), BUK (98.82%), BUNa (98.13%), BAUT (93.08%), BPD (98.92%), and BNO_3_ (98.02%). BPD gave the highest level of degradation for PAHs. The extents of degradation of TPH were as follows; BNK5 (94.50%), BNPK (94.77%), BUK (94.10%), BUNa (94.77%), BAUT (87.13%), BPD (95.35%), BNO_3_ (95.54%). TPH content in all treatments and controls were between 106 and 124.2 ppm by day 0. By day 56, the TPH content decreased and fell within 4.7 and 15 ppm. TPH content across all treatments and controls were not statistically significant at *P* < 0.05 using one way ANOVA and Tukey’s multiple comparison test. PAHs content in all treatments and controls were between 96 and 104.4 ppm by day 0. By day 56, PAHs content in all nutrient-amended treatments and controls decreased and fell within the range of 1.1 and 6.8 ppm. PAHs content across all treatments and controls were not statistically significant at *P* < 0.05 using one way ANOVA and Tukey’s multiple comparison test.

## Characteristics of bacterial isolates

A variety of bacteria were isolated from the nutrient-amended sediment samples during the 56-day bioremediation project, all of which were from genera of bacteria known to have the ability to degrade petroleum hydrocarbons. These isolates were fifty-nine in number, forty-nine of which were assigned tentative identities and belonged to the genera *Micrococcus*, *Staphylococcus*, *Pseudomonas*, *Citrobacter*, *Klebsiella*, *Corynebacterium*, *Bacillus*, *Rhodococcus*, *Alcanivorax*, *Alcaligenes*, *Serratia*, *Arthrobacter*, *Nocardia*, *Flavobacterium, Escherichia*, *Acinetobacter*, and *Proteus.* However, *Bacillus* appeared from the baseline to day 56, with *Pseudomonas*, *Rhodococcus*, *Alcanivorax*, and *Corynebacterium* being the dominant genera isolated.

Ten bacterial isolates could not be given tentative identities and were designated unidentified bacterial isolates. The diversity of bacterial isolates identified on days 0, 14 and 56 of the bioremediation experiment are presented in Tables 3, 4 and 5 while Table 6 shows the frequency of isolation of the bacteria identified in the study.

**Table 3:** Bacterial isolates identified during bioremediation of oil-polluted sediment on day 0

Isolate	Gram rxn	Oxidase	Citrate	MR	VP	Catalase	Indole	Gelatin	Urease	Motility	H_2_S prod.	Glucose F.	Gas. Prod.	AMP	COT	GEN	NAL	NIT	CHL	STR	TET	ERY	AUG	Tentative identity
BUNa I	+	−	+	+	−	+	+	+	−	−	−	+	−											*Micrococcus* sp.
BPD I	+	−	+	−	−	+	+	−	+	−	+	+	−											*Staphylococcus* sp.
BUK I	−	+	+	+	+	−	+	+	+	−	+	+	+	−	−	+	−	−	+	+	+	+	−	*Pseudomonas* sp.
BUK II	−	−	+	+	−	+	+	−	+	−	−	+	+	−	−	−	−	−	+	−	+	−	−	*Citrobacter* sp.
BUK III	−	+	+	+	−	+	+	−		−		+	+	−	−	−	−	−	+	−	+	−	−	*Pseudomonas* sp.
BUK IV	+	−	+	+	−	+	−	−	+	−		+	+											*Micrococcus* sp.
BNK5 I	−	−	+	+	+	+	−	+		−		+	+	−	−	+	−	−	+	+	−	−	−	*Klebsiella* sp.
BNPK I	+	−	+	+	+	+	+	+	+	−														*Corynebacterium* sp.
BNPK II	+	−	+	+	−	+	+	−	+	−	−	+	−											*Micrococcus* sp.
BNPK III	−	+	+	+	−	+	+	−	+	−	+	+	+	−	−	+	−	−	−	−	−	−	−	*Pseudomonas* sp.
BNO_3_ I	−	+	+	+	−	−	+	+	+	+	+	−	−	−	−	+	−	−	−	−	−	−	−	*Pseudomonas* sp.
BNO_3_ II	+	+	+	+	+	+	−	−	+	+	+	+	+											*Bacillus* sp.
BNO_3_ III	+	+	+	−	−	+	−	−	+	−	+	w	+											*Staphylococcus* sp.
BNO_3_ IV	−	+	+	+	−	−	+	−	−	−	−	+	+	−	−	−	−	−	−	−	−	−	−	*Pseudomonas* sp.

− Negative, + positive

*MR* methyl red, *VP* Voges Proskauer, *Amp* Ampiclox, *cot* cotrimoxazole, *Gen* Gentamycin, *Nal* Nalidixic acid, *nit* nitrofurantoin chl, *chl* Chloramphenicol, *Str* streptomycin, *tet* tetracycline, *ery* erythromycin, *w* weak reaction *NI* not identified

BNK5, Ammonium sulphate + dipotassium hydrogen phosphate (ratio 5:1) + 1 kg of sediment + 1 l of seawater + 20 ml of crude oil + 0.2 g PAH

BPD, Poultry droppings + dipotassium hydrogen phosphate (ratio 10:1) + 1 kg of sediment + 1 l of seawater + 20 ml of crude oil + 0.2 g PAH

BNO_3_, Ammonium nitrate + dipotassium hydrogen phosphate (ratio 5:1) + 1 kg of sediment + 1 l of seawater + 20 ml of crude oil + 0.2 g PAH

BUK, Urea + dipotassium hydrogen phosphate (ratio 5:1) + 1 kg of sediment + 1 l of seawater + 20 ml of crude oil + 0.2 g PAH

BNPK, Nitrogen, phosphorus and potassium fertilizer 20:10:10 (ratio 2:1:1) + 1 kg of sediment + 1 l of seawater + 20 ml of crude oil + 0.2 g PAH

BUNa, 1 kg of sediment + 1 l of seawater + 20 ml of crude oil + 0.2 g PAH

BAUT, 1 kg of heat killed sediment + 1L of heat killed seawater + 20 ml of crude oil + 0.2 g PAH

**Table 4:** Bacterial isolates identified during bioremediation of oil-polluted sediment on day 14

Iolate ID	Gran rxn	Oxidase	Citrate	MR	VP	Catalase	Indole	Gelatin	Urease	Motility	H_2_S prod.	Glucose F.	Gas. Prod.	AMP	COT	GEN	NAL	NIT	CHL	STR	TET	ERY	AUG	Tentative identity
BNPK 1	+	+	−	+	+	−	−	+	+	−	−	+	+											*Rhodococcus* sp.
BNPK 2	+	−	−	−	−	+	+	−	−	−	−	+	+											*Pseudomonas* sp.
BNPK 3	+	+	+	+	−	−	+	+	+	−	−	+	+											*Micrococcus* sp.
BNPK 4	−	+	−	−	−	−	−	−	−	−	−	w	−	−	−	+	−	−	+	−	+	−	−	*Alcanivorax* sp.
BNPK 5	−	+	+	−	+	−	+	+	+	−	+	+	+	−	−	−	−	−	−	−	+	−	−	*Alcaligenes* sp.
BNPK 6	+	+		+	−	−	−	−	−	−	−	+	+											NI
BNPK 7	+	+		+	+	−	−	−	+	−	+	+	+											NI
BNPK 8	−	−	+	+	−	−	−	+		+		+	+	−	−	+	−	−	+	+	−	−	−	*Serratia* sp.
BNPK 9	+	+	+	+	−	+		−		−	−	+	−											*Bacillus* sp.
BNO_3_ 1	+	+	+	+	+	−	−	−	+	−	−	+	−											NI
BNO_3_ 2	+	+	−	+	−		−	−	+	−	−	+	+											*Corynebacterium* sp.
BNO_3_ 3	+	−	−	−	−	−	+	−		−	+	+	+											*Rhodococcus* sp.
BNO_3_ 4	+	+	+	−	+		+	+	+	−	+	+	+											*Corynebacterium* sp.
BNO_3_ 5	+	−	+	+	+	−	−	+	+	−	+	+	−											*Arthrobacter* sp.
BNO_3_ 6	−	+	+	+	−	+	−	−	+	−	+	+	+	−	−	−	−	−	−	−	−	−	−	*Pseudomonas* sp.
BNK5 1	+		+	+	−	−	−	−	+	−	+	+	+											*Rhodococcus* sp.
BNK5 2	+	−	−	−	−	+	−	−		−	−	−	−											*Nocardia* sp.
BNK5 3	+	−	+	−	−	+	−	−	−	−	−	+	+											*Corynebacterium* sp.
BUNa 1	+	+	+			+		−	+	−	+	+	−											*Bacillus* sp.
BUNa 2	−	−	+	−	+	+	−	−	−	−	−	−	−											*Alcanivorax* sp.
BUNa 3	−	−	+	−	−	+	+	−	−	−	−	−	−	−	+	+	−	−	+	+	−	−	−	*Alcanivorax* sp.
BUK 1	+	+	+	−	+	−	−	−	+	+	+	+	+											*Nocardia* sp.

− Negative, + positive

*MR* methyl red, *VP* Voges Proskauer, *Amp* Ampiclox, *cot* cotrimoxazole, *Gen* Gentamycin, *Nal* Nalidixic acid, *nit* nitrofurantoin chl, *chl* Chloramphenicol, *Str* streptomycin, *tet* tetracycline, *ery* erythromycin, *w* weak reaction *NI* not identified

BNK5, Ammonium sulphate + dipotassium hydrogen phosphate (ratio 5:1) + 1 kg of sediment + 1 l of seawater + 20 ml of crude oil + 0.2 g PAH

BPD, Poultry droppings + dipotassium hydrogen phosphate (ratio 10:1) + 1 kg of sediment + 1 l of seawater + 20 ml of crude oil + 0.2 g PAH

BNO_3_, Ammonium nitrate + dipotassium hydrogen phosphate (ratio 5:1) + 1 kg of sediment + 1 l of seawater + 20 ml of crude oil + 0.2 g PAH

BUK, Urea + dipotassium hydrogen phosphate (ratio 5:1) + 1 kg of sediment + 1 l of seawater + 20 ml of crude oil + 0.2 g PAH

BNPK, Nitrogen, phosphorus and potassium fertilizer 20:10:10 (ratio 2:1:1) + 1 kg of sediment + 1 l of seawater + 20 ml of crude oil + 0.2 g PAH

BUNa, 1 kg of sediment + 1 l of seawater + 20 ml of crude oil + 0.2 g PAH

BAUT, 1 kg of heat killed sediment + 1L of heat killed seawater + 20 ml of crude oil + 0.2 g PAH

**Table 5:** Bacterial isolates identified during bioremediation of oil-polluted sediment on day 56

Isolate	Gram rxn	Oxidase	Citrate	MR	VP	Catalase	Indole	Gelatin	Urease	Motility	H_2_S Prod.	Glucose F.	Gas. Prod.	AMP	COT	GEN	NAL	NIT	CHL	STR	TET	ERY	AUG	Tentative identity
BUNa I	−	+	+	−	−	−	+	−	−	+	−	−	+	−	−	+	−	−	+	−	+	+	−	*Alcanivorax* sp.
BUNa II	+	+	+	−	+	−	+	−	+	−	−	−	+											*Rhodococcus* sp.
BUNa III	+	−	−	−	−	−	−	−	−	−	−	+	+											NI
BUNa IV	−	−	+	+	−	+	+	+	+	−	−	+	−	−	−	+	−	−	+	+	+	+	−	NI
BNPK I	+	+	+	−	−	−	+	−	−	+	−	+	+											NI
BNPK II	+	−	+	+	+	−	+	−	−	−	−	−	+											NI
BNPK III	+	+	−	+	+	−	+	−	−	+	+	+	−											*Flavobacterium* sp.
BPD I	−	−	+	−	−	−	+	+	−	−	−	−	+	−	−	−	−	−	−	−	−	−	−	*Alcanivorax* sp.
BPD II	+	+	+	+	−	−	+	−	−	+	−	+	+											*Staphylococcus* sp.
BPD III	+	+	−	+	−	−	+	−	−	−	−	+	+											*Escherichia* sp.
BUK I	−	−	−	+	−	+	+	+	−	−	−	−	−	−	+	+	–	–	+	+	+	+	–	*Alcanivorax* sp.
BUK II	+	−	+	+	−	−	+	−	−	+	−	−	−											*Acetobacter* sp.
BUK III	+	−	+	−	−	−	+	+	+	+	−	+	+											*Arthrobacter* sp.
BUK IV	+	−	+	+	+	−	+	+	−	−	−	−	+											*Rhodococcus* sp.
BUK V	+	+	+	−	+	+	+	−	−	−	−	+	−											*Corynebacterium* sp.
BNK5 I	−	−	+	−	+	−	+	+	−	+	−	+	+	−	−	−	−	−	−	−	−	−	−	*Proteus* sp.
BNK5 II	−	−	+	−	+	−	+	+	+	+	−	+	+	−	+	+	−	−	−	+	+	−	−	*Serratia* sp.
BNK5 III	+	+		+	+	−	+	+	−	−	−	+	+											*Rhodococcus* sp.
BNO_3_ I	+	−	+	−	+	−	+	−	−	+	−	+	+											*Bacillus* sp.
BNO_3_ II	+	+	−	−	+	−	+	+	−	−	−	−	+											NI
BNO_3_ III	+	−	−	−	−	−	+	−	−	+	−	−	−											NI
BNO_3_ IV	+	−	+	−	−	−	+	−	+	−	−	−	+											NI
BNO_3_ V	+	−		−	+	+	+	+	−	−	−	w	−											*Rhodococcus* sp.

− Negative, + positive

*MR* methyl red, *VP* Voges Proskauer, *Amp* Ampiclox, *cot* cotrimoxazole, *Gen* Gentamycin, *Nal* Nalidixic acid, *nit* nitrofurantoin chl, *chl* Chloramphenicol, *Str* streptomycin, *tet* tetracycline, *ery* erythromycin, *w* weak reaction *NI* not identified

BNK5, Ammonium sulphate + dipotassium hydrogen phosphate (ratio 5:1) + 1 kg of sediment + 1 l of seawater + 20 ml of crude oil + 0.2 g PAH

BPD, Poultry droppings + dipotassium hydrogen phosphate (ratio 10:1) + 1 kg of sediment + 1 l of seawater + 20 ml of crude oil + 0.2 g PAH

BNO_3_, Ammonium nitrate + dipotassium hydrogen phosphate (ratio 5:1) + 1 kg of sediment + 1 l of seawater + 20 ml of crude oil + 0.2 g PAH

BUK, Urea + dipotassium hydrogen phosphate (ratio 5:1) + 1 kg of sediment + 1 l of seawater + 20 ml of crude oil + 0.2 g PAH

BNPK, Nitrogen, phosphorus and potassium fertilizer 20:10:10 (ratio 2:1:1) + 1 kg of sediment + 1 l of seawater + 20 ml of crude oil + 0.2 g PAH

BUNa, 1 kg of sediment + 1 l of seawater + 20 ml of crude oil + 0.2 g PAH

BAUT, 1 kg of heat killed sediment + 1L of heat killed seawater + 20 ml of crude oil + 0.2 g PAH

**Table 6:** Frequency of isolation of different bacteria from sediment

Isolate	Frequency	Percentage occurrence (%)
*Micrococcus* spp.	4	6.8
*Staphylococcus* spp.	3	5.1
*Pseudomonas* spp.	7	11.9
*Citrobacter* sp.	1	1.7
*Klebsiella* sp.	1	1.7
*Corynebacterium* spp.	5	8.5
*Bacillus* spp.	4	6.8
*Rhodococcus* spp.	7	11.9
*Alcanivorax* spp.	7	11.9
*Alcaligenes* sp.	1	1.7
*Serratia* spp.	2	3.4
*Arthrobacter* sp.	1	1.7
*Nocardia* spp.	2	3.4
*Flavobacterium* sp.	1	1.7
*Escherichia* sp.	1	1.7
*Acetobacter* sp.	1	1.7
*Proteus* sp.	1	1.7
Unidentified bacteria	10	17.0

## Discussion and conclusion

Bioremediation of oil-polluted marine sediments was investigated in seven stirred-tank slurry bioreactors with appropriate amount of nutrient sources such as K_2_HPO_4_, NH_4_NO_3_, (NH_4_)_2_SO_4_, NPK, urea or poultry droppings to stimulate extant autochthonous marine bacteria. Physicochemical, total heterotrophic bacterial counts (THB), hydrocarbon utilizing bacterial counts (HUB), as well as gas chromatographic analyses were carried out on the nutrient-amended and control samples over a 56-day period as the experiment progressed. BUNa (unamended control) was composed of the sediment and indigenous bacteria only. The total heterotrophic bacterial (THB) count was 2.53 × 10^9^ cfu/g on day 0 and decreased to 9 × 10^8^ cfu/g on day 56. From this result, it was clear that the indigenous bacteria in the sediment were already acclimatized to hydrocarbons since there was also loss in TPH and PAHs in this control as bioremediation progressed. Odokuma and Dickson (2003) observed similar results.

The TPHs decreased from an initial 54.99748 to 6.26229 ng/μL, while the PAHs reduced from 98.27679 to 1.84442 ng/μL on day 56 with hydrocarbon chain lengths of C8 and C10 left for the TPHs. The unamended control (BUNa) contained populations of crude oil-degrading bacteria which increased with time with the concomitant depletion of hydrocarbons proving that indigenous bacterial communities in the hydrocarbon impacted-marine sediments have the natural capacity to degrade TPHs and PAHs since they could use crude oil components as a source of carbon and energy. Statistically, the rate of degradation of both TPH and PAHs in the unamended control and biostimulated treatments was not significant at *P* < 0.05 using one way ANOVA and Tukey’s multiple comparison test. This observation meant that biodegradation of crude oil hydrocarbons in the amended and control sediment slurries was taking place at similar rates. Similar observation was made by Rosenberg and Ron (1996) when they reviewed some of the case studies of bioremediation projects that took place shortly after the Exxon Valdez colossal oil spill. In one of such, the researchers used Inipol EAP22 oleophilic fertilizer to treat the oil-impacted shorelines. The researchers found out that C18:phytane ratio in the treated plots reduced during the summer of 1989 when the study was done. However, the control plots also showed a similar decrease in the ratio of hydrocarbons used as biodegradation index. Further statistical analysis showed that bioremediation effect was not significant at *P* = 0.05. Venosa et al. (1996) made similar observations when they investigated bioremediation of an experimental oil spill on the shoreline of Delaware Bay. They used a randomized block design to study the influence of biostimulation and bioaugmentation on the removal of crude oil in the contaminated sandy beach. High levels of oil biodegradation were seen in the untreated plots, and even though nutrient addition enhanced the rate of biodegradation, they concluded that there was no significant difference between plots treated with nutrients and those that were not. BAUT (heat-killed control) served to measure the effect of abiotic factors on biodegradation since all microbial life was removed by autoclaving the sediment slurry. In this treatment, TPHs reduced from 59.8377 to 14.9339 ng/μL, while the PAHs decreased from 98.0682 to 6.785 ng/μL on day 56. The rate of biodegradation was slightly less than that of the unamended sediment (BUNa) as well as the nutrient-amended sediments. The loss of hydrocarbons can be attributed to abiotic factors since a bioreactor was used and hence no leaching or evaporation of volatile fractions occurred (van Hamme et al. 2003). Invariably microbial activities coupled with abiotic factors (such agitation achieved using the stirrers in the bioreactors) in the sediment could be useful tools for remedial operations. In the amended slurries namely BPD, BUK, BNK5, BNPK and BNO_3_, it was observed that the THB and HUB counts increased in all five nutrient-enhanced sediments over the 56-day period resulting in corresponding hydrocarbon losses when compared to the heat-killed control that showed no microbial growth. Increases in bacterial counts (for both THB and HUB) in crude oil-polluted soils/sediments amended with organic and inorganic nutrient sources have been reported by other researchers. Roling et al. (2002) examined bacterial dynamics and crude oil degradation after biostimulation and found out that nutrient enhancement increased bacterial counts which correlated significantly with hydrocarbon attenuation. This same observation was made by several workers (Okpokwasili et al. 1986; Okpokwasili and Amanchukwu 1988; Okpokwasili and Odokuma 1994; Okpokwasili and James 1995; Okpokwasili and Ibe 1998; Margesin et al. 2003; Zucchi et al. 2003; Okpokwasili and Ibiene 2006; Okpokwasili and Oton 2006; Ruberto et al. 2006; Quatrini et al. 2008). In the present study, the THB and HUB counts obtained from the nutrient-amended slurries when compared with those from the oil-contaminated-unamended and heat-killed controls were statistically significant at *P* < 0.05. BNO_3_ had the highest THB count of 7.9 × 10^9^ cfu/g, which was closely followed by BPD (poultry litter amended slurry) which had a count of 4.4 × 10^9^ cfu/g on day 56. This increased count in BPD has been attributed to the diverse bacterial populations present in poultry droppings in addition to nutrients contained in it (Williams et al. 1999; Ijah and Antai 2003). This finding is in line with the report of El-Nawawy et al. (1992) that combining oily sludge with the application of inorganic fertilizers gave higher numbers of aerobic bacteria months after application when compared with untreated sediments. Amendment of the crude oil-polluted sediments with the various nutrient regimen stimulated more microbial proliferation in the sediments. The concentration of the crude oil-polluted sediments prior to nutrient enhancement was 3.35 ng/μL for TPHs with C8 (0.827 ng/μL), C10 (1.3096 ng/μL) and C12 (1.21016 ng/μL) chain lengths. The PAHs were naphthalene, fluorene, acenaphthylene, acenaphthene, phenanthrene, anthracene, fluoranthene, pyrene and chrysene with chrysene having the highest peak. The crude oil used in spiking the sediment had a TPH concentration of 6.07 × 10^5^ ng/μL, and PAH a concentration of 8.75 × 10^3^ ng/μL. The PAHs in the crude oil were the same as in the sediment but the TPHs had carbon chain lengths of C8–C26. With C8 having the highest concentration (1.79 × 10^5^ ng/μL). On day 0, the TPHs had a total concentration of 113.7922 on average for all treatments with C8–C14 hydrocarbons. The PAHs for all treatments had a concentration of 100.5153 ng/μL on average. On day 56 TPHs decreased appreciably to 5.2237 ng/μL in BPD; 6.3238 ng/μL in BUK; 5.5552 ng/μL in BNPK; 6.2622 ng/μL in BNK5. BNO_3_ had the highest degradation of 4.74559 ng/μL. For the PAHs, BPD showed the highest hydrocarbon loss (1.05032 ng/μL) when compared to the other treatments and the controls.

This may be due to the fact that nutrients were more in abundance in the poultry droppings than in the fertilizer and inorganic sources of nitrogen and phosphorus amended sediments. The hydrocarbon losses recorded in the biostimulated sediments slurries can be attributed to microbial activities which resulted in consumption of nitrogen and phosphorus added in the form of urea, NPK fertilizer, poultry droppings, and inorganic sources of nitrogen and phosphorus. Roling et al. (2004) reported that nutrient amendment over a wide range of concentration significantly improved crude oil degradation. The hydrocarbon utilizing bacteria isolated from the active bioreactors were *Pseudomonas* spp., *Serratia* sp., *Staphylococcus* spp., *Citrobacter* sp., *Micrococcus* spp., *Corynebacterium* spp., *Bacillus* sp., *Rhodococcus* spp., *Klebsiella* sp., *Flavobacterium* sp., *Alcanivorax* spp., *Alcaligenes* sp., *Nocardia* sp., *Arthrobacter* sp., *Escherichia* sp., *Proteus* sp., and *Acetobacter* sp. However, *Bacillus* appeared from the baseline to day 56, with *Pseudomonas*, *Rhodococcus*, *Alcanivorax* and *Corynebacterium* being the dominant genera isolated. The *Alcanivorax* spp. have been well documented as very important hydrocarbon degraders in marine sediments (Head et al. 2006; Yakimov et al. 2007; Rojo 2009).

These Gram-negative bacteria are peculiar as they cannot use carbohydrates and amino acids as growth substrates hence they are called ‘obligate hydrocarbonoclastic bacteria’ (OHCB). When grown on *n*-alkanes, however, they produce biosurfactants which have been shown to be glucose lipids. They use hydrocarbons almost exclusively as a carbon source. Recent works have revealed that the OHCB play a significant and global role in the natural cleansing of oil-polluted marine systems (Head et al. 2006; Yakimov et al. 2007; Peng et al. 2008; Alonso-Gutierrez et al. 2009; Gertler et al. 2009a, b; Wu et al. 2009; Qiao and Shao 2010; Ager et al. 2010; Obayori and Salam 2010; Nogales et al. 2011). Studies by Leahy and Colwell (1990) also revealed that the following bacterial genera contain well known species of hydrocarbon degraders in marine sediments; *Acinetobacter*, *Alcaligenes*, *Arthrobacter*, *Staphylococcus*, *Bacillus*, *Flavobacterium*, *Nocardia*, *andPseudomonas*, these bacteria were also isolated in this research project. Members of the Enterobacteriaceae family, e.g. *Klebsiella*, *Proteus*, *Serratia*, *Escherichia* isolated in this research corroborate the report of Prince (2005) which demonstrated them as hydrocarbon utilizers. Kasai et al*.* (2002) isolated *Flavobacterium* spp. from oil-polluted marine sediments capable of degrading aromatic hydrocarbons in crude oil. Said et al. (2008) isolated *Bacillus*, *Staphylococcus*, *Pseudomonas* and *Acinetobacter* spp. capable of degrading PAHs from polluted sediments. The study revealed that biostimulation of crude oil-impacted marine sediments with organic/inorganic sources of nitrogen and phosphorus encourages the proliferation of hydrocarbon utilizing bacteria. Bioremediation technique for removing petroleum hydrocarbons in sediments have been developed around strategies for delivering nutrients and altering the abiotic factors to optimize microbial activity and degradation of pollutants (Ayotamuno et al. 2006; Stroud et al. 2007). Bioremediation has long been applied as a remedial technology that is cost effective, ecologically friendly and efficient for the decontamination of crude oil-polluted sediments and soils (Kaplan and Kitts 2004; Nweke and Okpokwasili 2004; Quatrini et al. 2008). In this investigation, bioreactor-based treatment and amendment of crude oil-polluted sediments with poultry droppings, NPK and urea fertilizers, and inorganic sources of nitrogen and phosphorus caused more proliferation of crude oil-degrading bacteria and enhanced microbial degradation of crude oil in the sediment. A combination of NH_4_NO_3_, K_2_HPO_4_, and poultry droppings better enhanced hydrocarbon degradation than did the fertilizers urea and NPK alone. It was also observed that the unamended sediment which served as a natural attenuation control recorded appreciable hydrocarbon degradation. There was hydrocarbon loss in the heat-killed control signifying that abiotic factors could as well contribute to hydrocarbon attenuation in the environment. These results indicate that the marine sediment investigated is amenable to bioreactor-based bioremediation and that the extant autochthonous bacteria in the hydrocarbon-impacted Niger Delta sediments have the natural propensity to utilize hydrocarbons. Therefore, for effective bioremediation of petroleum hydrocarbon-impacted sediments, nitrogenous fertilizer (NPK and urea), poultry droppings and inorganic sources of nitrogen and phosphorus could be used. Further studies also need to be carried out in order to study in details the genetics of the hydrocarbon degrading bacteria in this Niger Delta marine sediments to ascertain the degradative genes/enzymes they posses.
